# Acrylamide content in starch based commercial foods by using high performance liquid chromatography and its association with browning index

**DOI:** 10.1016/j.crfs.2022.01.010

**Published:** 2022-02-20

**Authors:** Vandana Verma, Neelam Yadav

**Affiliations:** Centre of Food Technology, IPS, Faculty of Science, University of Allahabad, 21100, U.P, India

**Keywords:** Contaminants, Acrylamide, Browning index, French fries, Biscuits

## Abstract

The harmful effects of acrylamide (AA) are a major health concern for human beings. To find out the levels of AA content in commercial food products, 43 samples representing 3 important product categories (French fries, bakery biscuits, and branded biscuits) were procured from the local market in Allahabad, India. An assay of AA was done using HPLC-DAD. The LOD and LOQ for AA were 3.733 and 11.045 ng/μl, respectively. The AA recovery from ten standard solutions was 100.6 percent, indicating good extraction efficiency. Level of AA ranged between 144.35 and 781.17 μg/kg, 126.33–664.90 μg/kg, and 825.96–1143.15 μg/kg, in branded biscuits, bakery biscuits, and French fries, respectively. A strong and positive correlation of AA was found with HMF, a* value, BI and negatively with ΔE (P ≤ 0.01). It is concluded that a high browning index is significantly associated with AA content and can be used as a screening food to reduce the intake of AA in the diet.

## Highlights

• HMF and Acrylamide content in baked and fried RTE was analysed using HPLC-DAD/UV.
• HMF, color value, and browning index showed strong correlations with acrylamide.
• Browning index can be used as a screening tool for high acrylamide content food.

## Graphical abstract

**Figure:**
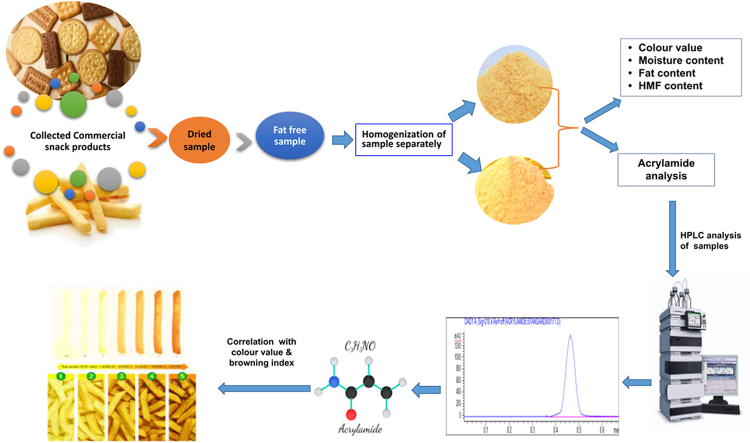

## Abbreviations

HMF: Hydroxymethylfurfural
AA: Acrylamide
LOD: Limit of detection
LOQ: Limit of quantiﬁcation
IARC: International Agency for Cancer Research
DAD: Diode array detection
HPLC: High pressure liquid chromatography
BI: Browning index
FARL: Food Analysis and Research Laboratory
SD: Standard deviation
S: Slope
SE: Standard error
CV: Correction value

## Practical application

This research will help the Indian food industry by modifying manufacturing measures to reduce the content of AA within foods. This will often make the customer conscious of consumable goods, that they should determine what amount can always be consumed every day to protect themselves from contaminants like HMF (5-hydroxymethylfurfural) and AA.

## 1 Introduction

Potato and wheat flour are basic ingredients of frequently consumed snack preparations, and both are rich sources of starch. They are stapled foods in the diets of most of the population and are rich sources of energy. French fries and biscuits are the most common food products made from potatoes and wheat flour and are consumed across the world by all age groups. In recent times, the most important vegetable crop in the world has been potatoes, followed by rice and wheat. Production of potatoes is greater in developing nations than in developed ones. Over the past 50 years, potato consumption has increased by 70% in both Asia and Africa (Su and Wang 2019; Wijesinha-Bettoniand Mouille 2019). The frying of potato strips depends on the transfer of heat through hot oil, resulting in the removal of water and the absorption of oil by the strips (Aguilera and Gloria-Hernadez, 2000), while the biscuits are subjected to another thermal treatment for baking. These processing methods are commonly used for the desired sensory or texture characteristics, to ensure microbiological safety, and to eliminate enzymatic activities. Maillard reactions, or non-enzymatic browning, are carried out during the thermal treatment of food products. This involves reducing sugar and carbonyl compounds with amino acids, peptides, and proteins. This chain reaction is complex, competitive, consecutive, and simultaneously generates many reactive intermediates and complex compounds such as AA, HMF, etc. (Zilic et al., 2020). EFSA 2015 (European Food Safety Authority). AA was discovered in a wide range of food items during the frying and baking processes, primarily potato chips, french fries, rice, coffee, tea, and baked foods. (Pedreschi et al., 2008; Gokmen et al., 2006). AA was listed as potentially carcinogenic for human beings by the International Agency for Cancer Research (IARC) in 1994 (IARC, 1994). In vivo and in vitro studies on AA toxicity confirm that it is a toxin that is both mutagenic and carcinogenic in humans and livestock, and can damage the nervous system (Dearfield et al., 1995). HMF is also present at a high level and is considered a marker of the degree of dehydration of Maillard and sugar reactions. It is produced by the occurrence of amino acids or proteins and by the acid-catalyzed thermal dehydration of fructose, sucrose, and glucose. The increased concentration of such markers, including HMF, is typically controlled by the severity of changes observed during the thermal processing of food (Rufian Henares et al., 2007). Based on the monitoring results of EFSA (2015), the European Commission released indicative values for different food products, but the current report of the Food Standard Agency (FSA) said that people in the UK have consumed greater than acceptable amounts of AA. In response, the European Commission is therefore releasing a new benchmark level for food in 2018 (European Commission, 2017). Similarly, an intermediate product, HMF has been specified by the commission of Codex Alimentarius for its highest limit as 40 mg/kg of honey to ensure that it has not experienced heat treatment during processing and is suitable for use (Alimentarius 2001). A study of the amount of AA in chosen food products has contributed to considerable interest in the occurrence of AA in food intake. It is commonly eaten all over the world in baked and fried foods, mainly in French fries, biscuits, and bakery items, which are almost exclusively consumed in India. The data on AA content in these potential foods is not available in India, hence the study was focused on investigating the risk of AA intake through these deep-fried and baked items. As regulation for HMF and AA has not yet been implemented in India, the current study has been undertaken with the hypothesis that it may occur at high content in a substantial number of samples.

## 2 Materials and methods

### 2.1 Food samples

Three different kinds of carbohydrate-rich products (n = 43), including French fries, branded biscuits, and bakery biscuits were randomly selected from different manufacturing batches. French fries were procured from the 16 various local restaurants of the Prayagraj, 13 branded biscuits from local shops, and 14 samples of biscuits from the local bakery shops of Prayagraj. The ground sample and its extract were stored at −10°C in a plastic screw-capped centrifuge tube until analysis. All the testing and HPLC analysis were done at the FARL (Food Analysis and Research Laboratory), Centre of Food Technology, University of Allahabad.

### 2.2 HPLC-DAD analysis of AA (Geng et al., 2008)

#### 2.2.1 Chemicals

AA standard was purchased by Sigma Aldrich (>99 percent) (Lot no. BCBV7937). Ultrapure water (Merk, Milli-Q model advantage A10) and HPLC grade reagents were used in the experiment, and syringe ﬁlters of 0.45 μm were purchased from Merk.

#### 2.2.2 Standard solution

Stock solutions of 1000 ppm AA are prepared and then diluted with ultrapure water to give several standard 10, 20, 30, 40, 50, 60, 70, 80, 90, and 100 ppm (ng/μl) solutions. For around 3 months, all the solutions were processed at 4C. Carrez 1 solution was prepared in 100 mL distilled water by dissolving 15 g of potassium hexacyanoferrate and Carrez 2 solution was prepared in 100 mL distilled water by dissolving 30 g of zinc sulfate.

#### 2.2.3 Sample preparation

One g of defatted sample was taken in the 15 ml of the centrifuge tube, afterward 10 ml water was added in the tube and centrifuged at 10^0^C, 10000 rpm for 10 min. The filtrate was transferred into another centrifuge tube and then the filtrate was added with 0.5 ml of both Carrez I and Carrez II solutions, mixed and again centrifuged at 10^0^C, 10000 rpm for 10 min, resulting in the supernatant was removed and evaporated in the water bath at 40^0^C for 15 min and ﬁltered with 0.45 μm cellulose acetate syringe ﬁlter paper.

#### 2.2.4 HPLC-DAD condition

The analysis was done by Agilent 1260 Infinity model of liquid chromatography system, 110 quaternary pumps (DEAB804078), 1260 thermostat column compartment (DEACN19021), Agilent injector with a loop of 50 μl volume, 1260 diode array, and multiple wavelength detector (DAD) (DEAAX02373). For the separation of the analyte, an isocratic elution pattern was adopted and the water & acetonitrile ratio (90:10) was used as the mobile phase. The method comprised of reverse, Zorbax column (SB–C18, 2.1 × 150 mm) was set at temperature 40 °C, flow rate 1 ml per min and detected at 210 nm wavelength by injecting 20 μl volume of solutions. For AA, the retention time was 0.463 min and the total run time was 15 min. Triplicate analysis of each solution was performed and the mean value of the results was used for calibration.

### 2.3 Color value

The Colour value of the French fries and biscuits was measured using Xrite (Grandville, MI, USA). The color attributes i.e. lightness (L*), Redness (a*), and Yellowness (b*) were recorded 3 times for each French fries, and the mean was calculated. L* ranges from 0 to 100, indicating luminance or lightness, and chromatic objects labeled as a* and b* range between −120 and 120, representing green to red and blue to yellow, respectively. The total color differences are referred to as the Euclidean distance (ΔE) calculated by using the following formula (Pathare et al., 2013).

$$\Delta \text{E }=\;\sqrt{\left(\left(\text{a*}\right)2+\;\left(\text{b*}\right)2\;+\;\left(\text{l*}\right)2\right)}$$

And the method used to measure the browning index (BI) was based on the CIE values of l*a*b* (Mohapatra et al., 2010):

BI =100 × (X -0.310.17)

where, X =(a∗ +1.75L)(5.645L + a∗ -3.012b*)

### 2.4 Moisture content

The moisture content of the sample was calculated by the (AOAC, 2005) method. The sample (5g) has been accurately weighed and transferred to a glass dish that is clean, dried, and weighed. During a hot air oven at 70^0^C, the contents were dried till a constant weight was attained and the reduction of weight was taken as moisture content after cooling and expressed in terms of percentage.

$$\mathrm{Moisture}\left(\mathrm{percent}\right)=\frac{\mathrm{Initial}\;\mathrm{weight}\;\mathrm{of}\;\mathrm{sample}\;-\;\mathrm{Weight}\;\mathrm{of}\;\mathrm{dried}\;\mathrm{sample}\;}{\;\mathrm{Initial}\;\mathrm{weight}\;\mathrm{of}\;\mathrm{sample}}\times 100$$

### 2.5 Fat content

The fat content of the samples was determined by the soxhlet extraction method (AOAC 2005). Five grams of the dried sample was taken into the thimble and was placed in the soxhlet apparatus with the petroleum ether (B.P. 60–80 °C) for 2-2 ½ hours. After evaporating the ether, the fat percentage was calculated by the subsequent expression.

$$Fat\left(percent\right)=\frac{Weight\;of\;the\;extract}{Weight\;of\;the\;sample}Χ100$$

Weight of ether extract = weight of extract with beaker-weight of the empty beaker.

### 2.6 Determination of HMF by spectrophotometer method (White, 1979)

Reagents: Carrez solution I - Dissolve 15 g of potassium ferrocyanide [K_4_Fe(CN)_6_.3H_2_O] in 100 ml ultrapure water. Carrez solution II - dissolve 30 g zinc acetate [Zn (CH_3_CO_2_) _2_∙2H_2_O] in 100 ml ultrapure water. Sodium bisulfite (NaHSO_3_) -Prepare 0.20 percent solution in ultrapure water.

Procedure: Take 5 g of homogenized food sample in 25 mL ultrapure water and pass all the solution to a 50 mL volumetric flask with a small amount of ultrapure water to wash the residue from the beaker. Add 0.5 mL of both Carrez I and Carrez II solution, mixed it, and make the volume with ultrapure water (one to two drops of alcohol might also be added that suppress the surface foam). Filter the solution and rejecting the primary 10 mL of filtrate. Transfer the 5 mL of filtrate in both two test tubes and then add 5 mL of ultrapure water into a sample and 5 ml of 0.20 percent bisulfite into the blank. Using the vortex mixer to blend properly. Measure the sample absorbance at 284 nm and 336 nm against the reference.

$$HMF\left(mg/kg\right)=\frac{\left[\left(A284-A336Χ74.8\right)\right]}{W}$$

where: W = weight of the sample (g)

$$A284,A336=\frac{Absorbance\;reading\;factor\;126\times 100\times 1000\times 100}{16830\times 1000}=74.87$$

126 = Molecular mass of HMF, 16830 = molar absorptivity of HMF at 284 nm.

### 2.7 Statistical analysis

The experimental result of triplicate measurements is expressed as mean ± SD (standard deviation). Data is subjected to one-way variance analysis (ANOVA) and calculated the signiﬁcance differences between means by Duncan’s multiple range test by using SPSS windows 16 and the signiﬁcance accepted at P ≤ 0.05 and ≤ 0.01.

## 3 Results and discussion

### 3.1 LOD (Limit of detection), LOQ (limit of quantification), recovery, and percent coefficient of variation

**Fig. 1:**
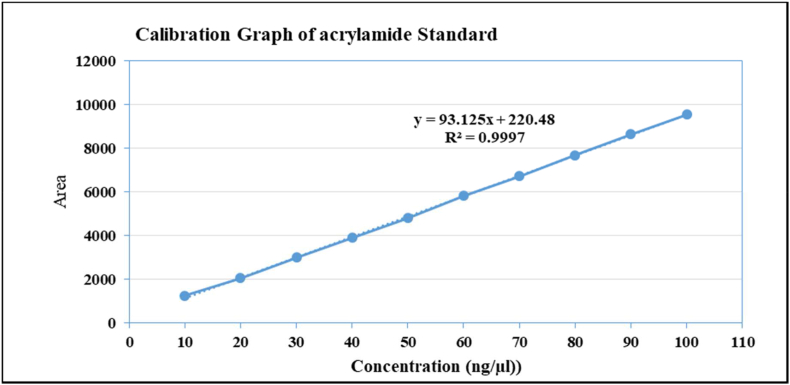
Calibration graph of acrylamide standard.

**Fig. 2:**
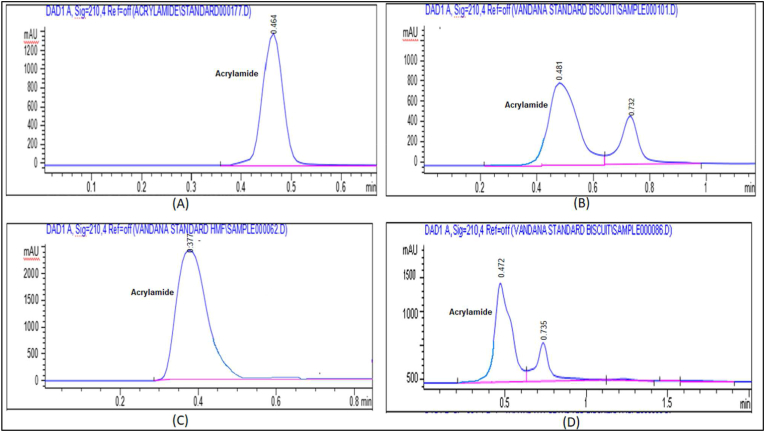
HPLC chromatograms of Acrylamide; (A) standard acrylamide, (B) Bakery biscuit, (C) French fries, (D) Branded biscuits.

For the detection and quantification of AA, the DAD (diode array detection) detector was used in HPLC. The linear calibration curve of ten concentration levels, with a regression coefficient (R^2^) 0.97773 and the peak area is correlated to the AA concentration linearly, as shown in Fig. 1. The LOD (limit of detection) and LOQ (limit of quantiﬁcation), SD (standard deviation of the response), slope of the calibration curve (S) Standard error (SE), and accuracy are given in Table 1. The LOD and LOQ were calculated by using the formula (3.3 × SD per S) and (10 × SD per S) respectively. The value of LOD and LOQ are 3.733 and 11.045 (ng per μl), respectively. Furthermore, the recovery percent were carried out by applying 10 known amounts of standard AA (10–100 ng per μl) to the blank water sample and run in HPLC. The recovery ranged is 98 percent to 110 percent with correction value (CV) less than 10 percent (Table 1). Using the above method for HPLC quantification, samples were extracted and all showed clear chromatograms peak at that retention time. Fig. 2 shows the chromatogram peak of AA in standard and sample samples. The difference between the processing conditions and chemical composition of the raw material was applied which may be the cause for the variance in the AA content between the various batches of a product category.

**Table 1:** Analytical data of the acrylamide standard solution analysed by HPLC-DAD.

S.N.	Concentrations (ng/μl)	Found Conc. (ng/μl)	Recovery (Percent)
1.	10	11.025	110.249
2.	20	19.668	98.340
3.	30	29.930	99.766
4.	40	39.628	99.071
5.	50	49.196	98.391
6.	60	60.066	100.110
7.	70	69.877	99.825
8.	80	80.044	100.055
9.	90	90.409	100.454
10.	100	100.158	100.158
**Correlation coefficient**	0.99973	**Mean recovery (%)**	100.642
**Accuracy**	100.64 ± 3.45	**SD**	3.455
**Slope**	94.748	**CV %**	3.433
**SE of intercept**	33.092	**LOD**	3.733
**SD of intercept**	104.645	**LOQ**	11.045

### 3.2 AA content in studied food sample

The AA level of 43 samples of 3 different kinds of carbohydrate-rich products, namely French fries, branded biscuits, and bakery biscuits sold in Allahabad district was determined the first time. AA levels in 13 samples of branded biscuits (H-1 to H-9), 14 samples of Bakery biscuits (A-1 to A-14), and 16 different restaurants of French fries samples F-1 to F-16 were calculated and given in Table 2. The result showed that AA content was high in 38–100 percent samples of three food groups with the highest in french fries with a mean value of 929.69 ± 4.55 followed by branded biscuits and bakery biscuits respectively (Table 3). European Commission (European Commission, 2017) has recommended a value of 500 μg per kg as indicative and 350 μg per kg as benchmark level for biscuits. Among the analysed biscuits sample 38% branded biscuits and 64.1 percent of bakery biscuits had AA content above the benchmark level of EFSA respectively. In branded and bakery category 15 percent and 7 percent sample were above the indicative value. Altogether 62 percent biscuits in branded and 36 percent sample from bakery section were having AA within acceptable range and rest were high. Table 3 shows that the mean value of AA was least in branded biscuits followed by bakery biscuits and French fries respectively. Wide variation in AA in branded biscuits may be due to the various favourable conditions of the manufacturing process and the availability of several products, such as wheat, oat, barley, corn, maize, rye, etc. Ingredients play an important role in the formation of AA since numerous cereals have varying quantities of asparagine and reducing the sugar required for the Maillard reaction. Mean AA content was found highest in the french-fried sample. This might be due to the presence of a higher amount of reducing sugar and asparagine in potato than cereal, which may increase the Maillard reaction leading to increased AA (Capuano and Fogliano, 2011). According to EC (European Commission), the indicative value and benchmark level of French fries are 600 μg per kg and 500 μg per kg respectively (European Commission, 2017). All 16 samples (100 percent) of French fries showed a concentration above indicative value as well as a benchmark value. The maximum level of AA was found in the sample of F6 and F16 sample of french fries i.e. 1143.16 ± 0.25 and 1057.52 ± 2.14 μg per kg respectively. AA has been characterized as an increased cancer risk for humans (Aladedunye et al., 2011), and based on previous studies HMF was found that harm human health leads to cytotoxicity to mucous membranes, skin, and high respiratory system. Reports of mutagenesis, chromosome abnormalities, human and animal carcinogenicity have also been reported due to AA toxicity (Glatt et al., 2005).

**Table 2:** Acrylamide content (μg/Kg) in French fries (F) branded biscuits (H) and bakery biscuits (A) samples.

French fries	RT (Min)	Acrylamide (μg per kg)	Branded biscuits	RT (Min)	Acrylamide (μg per kg)	Bakery biscuits	RT (Min)	Acrylamide (μg per kg)
**F1**	0.377	991.39 ± 0.92	**H1**	0.477	**781.17 ± 2.70**	**A1**	0.481	408.85 ± 3.19
**F2**	0.376	970.86 ± 9.40	**H2**	0.482	624.50 ± 1.00	**A2**	0.482	**126.33 ± 12.74**
**F3**	0.375	895.15 ± 1.62	**H3**	0.482	184.58 ± 11.37	**A3**	0.468	567.26 ± 0.49
**F4**	0.378	850.02 ± 0.38	**H4**	0.479	322.72 ± 8.11	**A4**	0.467	494.41 ± 1.62
**F5**	0.379	885.39 ± 0.43	**H5**	0.475	188.27 ± 0.00	**A5**	0.468	403.95 ± 1.61
**F6**	0.381	**1143.16 ± 0.25**	**H6**	0.461	**144.36 ± 17.26**	**A6**	0.467	290.65 ± 0.20
**F7**	0.393	884.91 ± 0.99	**H7**	0.477	**725.63 ± 1.39**	**A7**	0.47	376.05 ± 11.58
**F8**	0.38	936.18 ± 7.36	**H8**	0.475	504.21 ± 3.83	**A8**	0.469	327.79 ± 0.57
**F9**	0.391	836.75 ± 13.87	**H9**	0.489	296.73 ± 8.72	**A9**	0.47	294.84 ± 1.30
**F10**	0.388	896.11 ± 11.78	**H10**	0.476	366.40 ± 7.08	**A10**	0.458	429.11 ± 5.60
**F11**	0.385	825.96 ± 1.82	**H11**	0.469	198.38 ± 11.12	**A11**	0.468	481.14 ± 4.38
**F12**	0.389	949.59 ± 5.48	**H12**	0.481	**148.33 ± 12.22**	**A12**	0.466	334.62 ± 7.83
**F13**	0.386	914.76 ± 0.46	**H13**	0.472	318.82 ± 26.36	**A13**	0.466	369.91 ± 6.73
**F14**	0.379	908.71 ± 0.28	5 sample (38 percent) showed a concentration above 350 μg/kg Benchmark level.			**A14**	0.462	**664.91 ± 0.04**
**F15**	0.396	928.67 ± 0.06				9 sample (64 percent) showed a concentration above 350 μg per kg Benchmark level		
**F16**	0.380	**1057.52 ± 2.14**						
All sample (100 percent) showed a concentration above 500 μg per kg benchmark level								

All values (Average ± Standard deviation of n = 3 independent samples) are calculated by excel.

**Table 3:** Acrylamide content (μg per kg) of some commercial food products.

CODE	No. of sample (n)	Moisture (Percent)	Fat (Percent)	a*	BI	ΔE	HMF (mg per kg)		Acrylamide (μg per kg)	
							Mean	Range	Mean	Range
**French fries**	16	32.41	14.01	12.46	125.30	64.56	9.19	5.65–71.81	929.69 ± 4.55^b^	825.96–1143.15
		±6.77	±6.93	±4.6	±50.95	±8.9	±23.70^b^			
**Branded biscuits**	13	6.07	16.67 ± 8.99	11.42	112.15	67.29	6.37	2.81–9.73	369.54 ± 7.42^a^	144.358–781.173
		±1.47		±2.26	±24.83	±16.49	±0.2.54^a^			
**Bakery biscuits**	14	6.08	14.08	15.17	99.39	81.04	9.80	3.26–19.36	397.84 ± 4.22^a^	126.33–664.90
		±2.26	±5.76	±2.50	±32.18	±5.89	±4.82^a^			

All values (Average ± Standard deviation of total samples of food groups) and ^a-b^ superscript with different alphabet shows signiﬁcance difference at P ≤ 0.05 are calculated by excel.

The mean value of AA in French fries, branded biscuits, and bakery biscuits were 929.69, 369.54, and 397.84 μg per kg, respectively. The mean and median AA value of french fries were 929.69 μg per kg and 911.73 μg per kg were higher than branded biscuits (369.54 and 318.82 μg per kg) and bakery biscuits (397.84 and 390 μg per kg) respectively and also agree with data recorded in Europe and other countries (Table 3). Statistically, they were significant differences between the mean value of AA in French fries, branded biscuits, and bakery biscuits (p > 0.05). However, no significant differences were found in the AA levels of biscuits, French fries, and bakery biscuits that belong to various brands and types (Table 3). Within the food groups percentage of samples with AA concentration above the EFSA-recommended indicative values was higher in French fries (100 percent) than the branded biscuits (38 percent) and bakery biscuits sample (64 percent) (European Commission, 2017). Numerous studies on AA levels in potato products in several countries have been conducted. In Iran, the AA contt study of Iranian potato and maize brands revealed that the level of AA was between 244 and 1688 μg per kg in potato products and between 30 and 410 μg per kg in corn products (JECFA 2011; JECFA 2006; EFSA 2012 and Oroushaki et al., 2010). Similar results were reported by Shamla and Nisha, (2014) where the concentration of AA in four different snack group ranged between 82.0 and 4245.6 μg per kg 46.2–2431.4 μg per kg, 24.8–1959.8 μg per kg, and 14.7–1690.5 μg per kg for potato chips, jack chips, sweet plantain chips, and plantain chips respectively (Shamla and Nisha, 2014). Some more studies are in the favour of our result, Boyaci Gunduz et al. (2017) also reported the same result, where AA levels by a GC/MS process with bromine derivatization, 90 commercial samples of crackers, biscuits, and baby biscuits sold in Turkey were determined. The mean AA values were 604 μg per kg in crackers, 495 μg per kg in biscuits, and 153 μg per kg in baby biscuits. As hypothesized in our study a very high number of samples with AA content above the benchmark level of the European commission was observed in our study. Effective implementation of regulators by FSSAI may initiate the required processing steps to mitigate the AA formation in baked and fried foods. Using high reducing sugar in carbohydrate-rich formulations increases the AA content at high temperatures. In the preparation and processing of biscuits, this is related to the components of mixtures and the number of carbohydrates that are responsible for the high of AA (Sanny et al., 2012; Verma and Yadav, 2020). The additives containing acids and materials like sodium bicarbonate may play a role in reducing the pH leading to less formation of AA (Bassama et al., 2010; Eriksson, 2005).

### 3.3 Moisture, fat, HMF, browning index (BI), and color value of studied samples

The compositions of 43 samples of three food groups are given in Table 3. As we know that HMF was the intermediate compound of the Maillard reaction and this is very much responsible for AA formation in the cooked food therefore determination of HMF levels in foods seems to be necessary. The safe level of HMF has not been reported in various food products, however, the suggestion is to focus on many works from which accumulation of data about such food products may be reviewed so guideline is framed for setting up of maximum permitted levels of HMF in commonly available foods (Pastoriza et al., 2017).

**Fig. 3:**
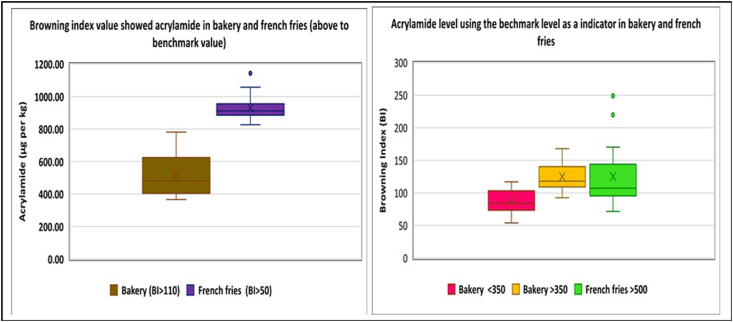
Box-and -whisker plot of browning index (BI) and acrylamide (benchmark level) using as indicator in biscuits and French fries.

The HMF, BI content, and a* value in French fries were found higher than both kinds of biscuits whereas ΔE was lowest in French fries. A strong and positive correlation of AA was found with HMF, a* value, BI and negatively with ΔE (P ≤ 0.01). Similarly, HMF content was strongly correlated with BI and a* value (P ≤ 0.01) (Table 4). A similar finding was obtained by Pathare et al., (2012), Pedreschi et al., (2005) and Mesias et al., (2020), who stated linear correlation (Mean R^2^ = 0.9569) of HMF with a* values for fried potato slices. Another finding from this study is that the parameter BI appears to differentiate samples according to the AA level using the benchmark as a threshold (500 μg/kg) that show100 percent data french fries and >50 percent data of bakery products have an AA value above the benchmark level. Therefore, BI can be a very effective indicator and can be developed as a screening tool by eliminating dark-colored or toasted snacks for avoiding exposure or consumption of AA in food (Fig. 3). However, the high levels of AA detected in some of the samples show that these criteria are not enough since the perception of BI is a subjective process.

**Table 4:** Correlation of HMF content and Acrylamide with moisture, fat, a^b^ value, ΔE and browning index.

Parameter	French fries				Biscuits			
	Acrylamide		HMF		Acrylamide		HMF	
	Rho Pearson (r)	*P value*	Rho Pearson (r)	*P value*	Rho Pearson (r)	*P value*	Rho Pearson (r)	*P value*
**Moisture**	-.185	.493	-.690^a^	.003	.403^b^	.037	.426^b^	.027
**Fat**	.006	.983	.519^b^	.039	-.189	.346	-.195	.329
**a**	.518^b^	.040	.928^a^	.000	.690^a^	.000	.812^a^	.000
**BI**	.523^b^	.037	.941^a^	.000	.756^a^	.000	.606^a^	.001
**ΔE**	-.598^b^	.015	-.413	.112	.351	.073	.442^b^	.021
**HMF**	.543^b^	.030			.735^a^	.000		

N=43

a Correlation is significant at the 0.01 level (1-tailed).

b Correlation is significant at the 0.05 level (1-tailed).

The method was not well understood so, it is impossible to debate the significance of ingredients. But it is identified that the sorts of flour and potato, sorts of ingredient, and variation in frying and baking time and temperature can accelerate or inhibit the AA formation. In the present study, large variations within the AA contents of various brands and kinds of food groups were recorded. Similar outcomes have been reported by other research teams (Olmez et al., 2008). This variation is often because of the ingredient and processing treatment in several brands and kinds of food samples as AA formation is correlated with these aspects. Differences within the staple composition like reducing sugar, free asparagine and food product formulations methods like pH, moisture, heat (more than 120^0^C), and duration might be accountable for the formation of AA as stated by other researchers (Friedman and Levin, 2008). Industries should follow correct recipes for decreasing AA in food products, or there would be a chance of risk factor for consumers to be affected by different diseases. Depending upon the products, the manufacturer should use fresh materials to control AA levels because the reducing sugar increased in stored potatoes (at low temperatures) and the presence of asparagine in these potatoes react with reducing sugar and may lead to the formation of AA (Stadler et al., 2004). Due to the risk of AA, its production in baked and fried products should be reduced in food and maybe a health concern. Industries should produce AA-free products by taking the necessary measures or using potato varieties having a low content of reducing sugar. If stored potatoes are used for processing, they should be stored at 8–12^0^C to prevent the formation of reducing sugar (Ezekiel et al., 2007bib_Ezekiel_et_al_2007). The potato and fried foods should be standardized for golden-yellow, not for brown (Elmore et al., 2015). The frying condition should not be exceeded 175^0^C and oven baking temperature should not be exceeded 200^0^C and water should be well-drained off from soaked potatoes (Ahrne et al., 2007). For baked products like biscuits, the suggested measure could be standardized of baking temperature, microwave baking or combination, replacement of reducing sugar, using flours have a low amount of asparagine and carbohydrate. These steps can reduce the AA content in baked food products (Surdyk et al., 2004).

## 4 Conclusion

A quality addition technique was used to measure AA in heat-processed food samples. All these samples consist of carcinogenic AA contaminant 1143.15 μg per kg with the highest content in French fries. Results of the study confirmed that AA is usually found high in fried and baked food products which are commonly consumed by India. All samples of French fries and more than 50 percent of baked biscuits contain AA levels above the indicative value of European Standards. It should alarm both industries and regulatory authorities. Studies showed that the browning index can be used as a measuring index and efforts should be made to avoid excess browning. This important research will help the Indian food industry by modifying manufacturing measures to compress the content of AA within the foods. This will often make the customer conscious of consumable goods and that they should determine what amount can always be consumed every day to protect themselves from contaminants like HMF and AA. Mitigation and control of the AA program should be introduced by official bodies and food manufacturers in India. FSSAI (Food Safety and Standards Authority of India) has already initiated steps to formulate for framing standard. Guideline for AA and this study will provide useful information.

## Author's contributions

Both authors made important contributions and follow to its publication. The contribution of the authors was as follows: Neelam Yadav: Supervision, Conceptualization, Visualization, Reviewing and Finalizing. Vandana Verma: Methodology, Software, Validation, Data curation, Writing- Original draft preparation, Editing, Investigation.

## Funding

The research did not fund by any agencies and resources.

## Availability of data

This article contains all of the data collected during this study, as well as supplementary material that is kept confidential between the publisher and the author.

## Author details

Vandana Verma and Neelam Yadav, Centre of Food Technology, IPS, Faculty of Science, University of Allahabad, Prayagraj, U.P-211002*.*

## CRediT authorship contribution statement

**Vandana Verma:** Methodology, Software, Validation, Data curation, Writing – original draft, Editing, Investigation. **Neelam Yadav:** Supervision, Conceptualization, Visualization, Reviewing and Finalizing.

## Declaration of Competing Interest

The authors declare that they have no known competing financial interests or personal relationships that could have appeared to influence the work reported in this paper.
